# 回生口服液用于非小细胞肺癌围手术期抗凝治疗专家共识（2016版）

**DOI:** 10.3779/j.issn.1009-3419.2016.11.01

**Published:** 2016-11-20

**Authors:** 清华 周, 俊峰 刘, 晓光 杨, 合林 张, 繁义 孔, 国臣 王, 宪利 孟, 志全 陈, 立军 柳, 潞 李, 雄志 吴, 杨 郭

**Affiliations:** 1 610041 成都，四川大学华西医院肺癌中心 Lung Cancer Center, West China Hospital, Sichuan University, Chengdu 610041, China; 2 050011 石家庄，河北医科大学第四医院/河北省肿瘤医院 Forth Hospital of Hebei Medical University/Tumor Hospital of Hebei Province, Shijiazhuang 050011, China; 3 056001 邯郸，邯郸市中心医院 Handan Central Hospital, Handan 056001, China; 4 050000 石家庄，河北医科大学第二医院 The Second Hospital of Hebei Medical University, Shijiazhuang 050000, China; 5 061001 沧州，沧州市中心医院 Cangzhou Central Hospital, Cangzhou 061001, China; 6 063000 唐山，河北联合大学附属医院 North China University of Science and Technology Affiliated Hospital, Tangshan 063000, China; 7 050055 石家庄，河北省人民医院 Hebei General Hospital, Shijiazhuang 050055, China; 8 300040 天津，天津市肿瘤医院，肿瘤研究所/天津医科大学肿瘤医院 Cancer Hospital/Institute, Tianjin Medical University, Tianjin 300040, China

原发性肺癌是我国发病率和死亡率增长最快，对人群健康和生命威胁最大的恶性肿瘤，也是导致我国人群癌症相关死亡的首要原因^[1, 2]^。非小细胞肺癌占全部肺癌患者的85%左右。迄今为止，手术治疗仍是早期非小细胞肺癌的最佳治疗方法^[1]^。静脉血栓栓塞症（venous thromboembolism, VTE）是肺癌最常见的并发症并影响预后^[2]^，已成为导致肿瘤患者死亡的第2位原因^[3]^。美国临床肿瘤学会（American Society of Clinical Oncology, ASCO）在2010年颁布的"恶性肿瘤患者静脉血栓防治指南"表明，癌症患者，特别是接受大手术或全身性治疗的患者，静脉血栓栓塞的风险显著增加。国内一项单中心VTE患者临床资料分析^[4]^显示，9年内201例VTE患者中有28.4%基础疾病为肿瘤；若无有效的预防措施，行外科手术的肿瘤患者中，深静脉血栓形成发生率高达40%-80%，肿瘤大手术患者中肺栓塞的发生率为4%-10%。上海胸科医院陆舜教授报道1, 001例肺癌患者手术后的1个月、3个月、6个月、12个月和30个月内VTE的发生率分别为2%、3%、4%、5%和5.3%^[4]^。另有回顾性研究^[5]^发现肺部恶性肿瘤（大部分为原发性支气管肺癌）患者肺部手术后7天内VTE发病率高达7.4%。故对肺癌患者进行围手术期静脉血栓防治尤为必要。

目前用于防治VTE的药物主要是低分子肝素，有研究^[6]^表明肿瘤化疗患者使用低分子肝素可以提高化疗疗效，但该药需要进行皮下注射；口服药包括利伐沙班、达比加群、阿哌沙班等，其对于恶性肿瘤患者VTE的防治效果有待进一步临床验证^[7-10]^。

“活血化瘀”是中医治疗肿瘤的重要手段。回生口服液为“活血化瘀”治疗肿瘤的传统代表方剂，抗肿瘤的同时表现出明确的抗凝和抗血栓作用。回生口服液的主要成分中水蛭、虻虫、红花的提取物有改善凝血功能、抗血小板（blood platelet, PLT）聚集的作用^[11]^。

## 1 体外实验证据

组方的活血化瘀研究发现回生口服液可以改善急、慢性血液高凝状态，用角叉菜胶造成大鼠血栓模型，回生口服液灌胃组FDP及D-二聚体的检测值均较对照组明显降低（24 h检测值除外）（*P* < 0.05），PLT检测值均较对照组明显降低（*P* < 0.05），肺脏、肝脏及肠系膜组织内微血栓的数量均较对照组明显减少（*P* < 0.05）^[12]^，对荷Lewis肺癌小鼠，回生口服液能显著抑制Lewis肺癌成瘤性，并减少肺转移灶和肺栓塞形成^[13]^。

## 2 肺癌患者围手术期抗凝治疗

Gu等^[14]^对就诊于5家医院的104例原发性支气管肺癌手术患者开展研究发现，试验组（患者接受手术治疗和回生口服液）和对照组（患者仅接受手术治疗）在PLT、D-二聚体指标上有显著性差异。在河北省五家中心开展的“回生口服液对原发性支气管肺癌围手术期凝血功能影响的研究”（注册号ChiCTR-TRC-13003325）中发现，该药对Ⅰ期-Ⅱ期肺癌手术患者的PLT、FIB、D-二聚体等指标均有明显改善。结合我国国情，将传统中药与手术治疗相结合，对肺癌围手术期患者的抗凝抗血栓治疗进行规范并达成共识是提高手术疗效、改善预后、延长中位生存时间、降低死亡率的有效办法。对此，专家建议在肺癌围手术期抗高凝、抗血栓的预防和治疗过程中：①若无出血，术前，入院后尽早使用回生口服液至术前1天。服用方法：口服，20 mL/次，2次/d。②排除出血，术后3天开始继续服用回生口服液至术后1个月。服用方法：口服，20 mL/次，3次/d。

## 3 肺癌患者术后（出院后）长期维持治疗

近年来，动物实验和流行病学调查均发现，凝血指标包括血浆凝血酶原时间、凝血酶原活动度、国际标准化比率、活化部分凝血活酶时间、纤维蛋白原、D-二聚体、PLT计数等均是影响肺癌预后的重要因素，尤其是机体高凝状态与术后肿瘤转移增强明显相关^[15]^。一项包含7项研究共计1, 377例肺癌患者的*meta*分析研究^[16]^显示，D-二聚体水平越高，患者生存时间减少的风险越大，HR=1.12（1.02-1.23）；另一项针对78例肺癌患者（60例非小细胞癌和18例小细胞癌）术前D-二聚体水平的研究^[17]^表明，与低D-二聚体水平（≤0.65 μg/mL）比较，高D-二聚体水平（> 0.65 μg/mL）减少患者生存期。同时，关于纤维蛋白原水平与肺癌预后的研究，包含52项研究纳入15, 371例肺癌患者的结果^[18]^显示，术前高的血浆纤维蛋白原水平降低患者无病生存时间，HR=1.69（1.48-1.92），而针对567例可手术的非小细胞肺癌患者的研究^[19]^发现，术前高的纤维蛋白原水平，分别增加疾病进展风险，HR=1.49（1.07-2.05）和死亡的风险，HR=1.64（1.06-2.53）。此外，包含12项研究共计5, 884例肺癌患者的PLT计数水平表明^[20]^，高的血小板计数水平，增加了总生存时间减少的风险，HR=1.74（1.39-2.19），同时Yu等^[21]^、Liu等^[22]^针对肺癌患者术前PLT计数水平的研究显示，术前高的PLT计数水平分别增加非小细胞癌进展风险，HR=1.57（1.02-2.45）和淋巴结转移的风险。

Gu等^[14]^报道回生口服液可以有效降低肺癌患者围手术期PLT计数、血浆D-二聚体和纤维蛋白原含量。有研究^[23]^表明，回生口服液对随访期（6个月-24个月）卵巢癌患者有确切的康复治疗效果。回生口服液对肺癌术后患者的远期生存影响尚无明确结论，不过根据Cochrane协作组发表的综述结果来看^[24-26]^，恶性肿瘤抗凝治疗目前较为明确的结果是有效减少了VTE的发生，但恶性肿瘤和血液高凝相互影响，涉及炎症反应、血管内皮损伤、凝血启动、PLT活化、肿瘤细胞粘附聚集等多种作用环节，相关的细胞因子、蛋白种类众多，以PLT活化为例，大量研究发现肿瘤转移与之密切相关^[27-29]^。此外，具有抗炎、抑制PLT活化作用的阿司匹林可改善多种肿瘤的预后情况^[30-32]^。

## 4 安全性

回生口服液在临床使用中未见导致出血的报道，但该药含有大量活血化瘀成分，专家建议，在患者已有活动性出血或者肝功Child-pugh分级为C级者，慎用回生口服液。

## 5 小结

建议肺癌患者术后应尽早开展抗凝治疗，回生口服液为肺癌患者术后长期、足量抗凝提供了一种新的用药选择，可作为肺癌围手术期抗凝治疗的选择之一。其在短期内活血化瘀方面的作用及其远期对肿瘤复发、转移的影响，值得在临床进一步探讨和推广。随着后续研究结果的不断增多，专家委员会将随之适时更新本共识。
